# Death from Hepatic Abscess Bursting into the Pericardium

**Published:** 1845-09

**Authors:** R. Allan

**Affiliations:** Staff-Surgeon, Port Louis, Mauritius


*Death from Hepatic Abscess bursting into the Pericardium.* By R. Allan, Esq., Staff-Surgeon, Port Louis, Mauritius—Cumia, aged 35, native of Bombay, had been in Mauritius one year, working as a field laborer, when he came into the Immigration Depot, on the 21st of December, 1844, for the purpose of entering into a new engagement, having walked seven or eight miles on that day. He remained in apparent good health until six o'clock on the morning of the 26th, when he began to complain of pain at the pit of the stomach, and died at half-past 10 A.M.

*Sectio cadaveris twenty-four hours after death.*—About two pints of reddish pus and serum within the pericardium, the entire of which membrane was slightly inflamed. On laying the pericardium freely open, thick yellowish-green pus was seen oozing from an aperture large enough to admit the finger, which led through the diaphragm into abscess in the smaller lobe of the liver, capable of containing a pint of fluid.

It is probable that the pus of the hepatic abscess had been oozing into the sac of the pericardium during some hours before death, causing inflammation, and then annihilating the heart's action by pressure.

Messrs. Rogers and Powell, surgeons, were present with me at the dissection, and we have forwarded the parts to the Military Museum at Chatham.—*Ibid.*

